# Restoring Global Gene Regulation through Experimental Evolution Uncovers a NAP (Nucleoid-Associated Protein)-Like Behavior of Crp/Cap

**DOI:** 10.1128/mBio.02028-21

**Published:** 2021-10-26

**Authors:** Sophia A. H. Heyde, Pernille O. Frendorf, Ida Lauritsen, Morten H. H. Nørholm

**Affiliations:** a Novo Nordisk Foundation Center for Biosustainability, Technical University of Denmark, Kongens Lyngby, Denmark; Korea Advanced Institute of Science and Technology

**Keywords:** cyclic AMP receptor protein, experimental evolution, global regulatory networks, supercoiling, topoisomerases, transcription factors

## Abstract

How do hierarchical gene regulation networks evolve in bacteria? Nucleoid-associated proteins (NAPs) influence the overall structure of bacterial genomes, sigma factors and global transcription factors (TFs) control thousands of genes, and many operons are regulated by highly specific TFs that in turn are controlled allosterically by cellular metabolites. These regulatory hierarchies have been shaped by millions of years of evolution to optimize fitness in response to changing environmental conditions, but it is unclear how NAPs and TFs relate and have evolved together. Cyclic AMP (cAMP) receptor protein (Crp) is the paradigmatic global TF in Escherichia coli, and here we report that mutations in the *topA* gene compensate for loss of cAMP, showing that the interplay between Crp and the supercoiling status of promoters is key to global stress response. Furthermore, we observed an effect of apoCrp on gene expression in the absence of its effector cAMP. This provides support for the proposed NAP-like role for Crp, suggesting that it represents an intermediate point in the evolution of a ligand-controlled TF from a NAP.

## INTRODUCTION

Regulation of gene expression is central to the adaptability of bacterial cells in response to changes in the environment. In the model bacterium Escherichia coli, the cyclic AMP (cAMP) receptor protein (Crp) plays an overarching role (1). Modulating the expression of hundreds of genes (2) in the E. coli genome, mostly involved in carbon metabolism (1), Crp is a global transcription factor (TF) that can act both as an activator and as a repressor upon binding its ligand cAMP. Crp is one of the most thoroughly studied TFs and provided one of the first examples that gene regulation is more complex than suggested by the Jacob-Monod operon model (3). The E. coli genome contains several thousand putative binding sites for Crp (4), of which only a fraction are known to affect promoter activity. Combined with observations of unspecific DNA binding affinity and bending of DNA by ∼90° upon binding (5) and cooperative DNA binding and condensation by apoCrp (6, 7), an additional role of Crp as a nucleoid-associated protein (NAP) involved in the global organization of the bacterial chromosome has been hypothesized (3, 8, 9).

The genome structure of E. coli is under the control of a homeostatic network comprising DNA topoisomerases, abundant NAPs, and components of the transcriptional machinery, coordinating both genes mediating stress adaptation and genes of the central metabolism. The interplay between global and local TFs is particularly important when bacteria experience stress that calls for a substantial reset of the transcriptional machinery. For example, temperature upshift from 30°C to 42°C leads to rapid induction of more than 20 heat shock proteins (HSPs), including the molecular chaperones DnaK, DnaJ/GrpE, and GroEL/GroES, as well as multiple proteases (10). HSPs are essential for survival of the cell, since they protect other proteins from thermal inactivation and reactivate aggregated proteins. Transcription of HSP genes is positively controlled by the sigma factor σ^32^, encoded by the *rpoH* gene (11).

DNA supercoiling is an important sensor for changes in the physical environment. σ^32^ expression is sensitive to changes in the supercoiling state of the bacterial chromosome (12) and is actively controlled by DnaKJ and GroEL/S, while σ^32^ levels are reduced via degradation mediated by the FtsH protease (13). The *topA* gene, encoding E. coli DNA topoisomerase I (TopoI), is also controlled by a σ^32^-dependent heat shock promoter (14). TopoI relaxes negatively supercoiled DNA, while its antagonist DNA gyrase introduces negative supercoiling into relaxed DNA (15). During heat shock, relaxation of DNA is facilitated by a tight interplay of both enzymes, which is essential to counterbalance the DNA unwinding that occurs at high temperatures in E. coli (16, 17).

The DNA topology in promoter regions directly influences gene expression. Biosynthetic genes are preferentially transcribed under conditions of high negative supercoiling, whereas transcription of catabolic genes involved in energy production are activated under conditions of DNA relaxation (18). The effective level of supercoiling is thereby determined by competition between NAPs, such as HU, H-NS, and Fis, as well as RNA polymerases, balancing the compaction and availability of DNA (19). Both Fis and Crp regulate the expression of numerous genes, needed for carbon and nucleic acid catabolism, and impact DNA topology in a growth-phase-dependent manner (1, 20). While the cAMP-Crp complex activates expression of the *gyrA* gene and increases the negative supercoiling level of reporter plasmids, Fis inhibits DNA gyrase and tightly regulates *topA* expression (20, 21). Expression of both *crp* and *fis* responds to changes in DNA topology, with *fis* expression being maximal at high levels of negative supercoiling and *crp* transcription being achieved by oscillation of multiple regulatory nucleoprotein complexes in response to the physiological state of the cell, tightly linking the global regulatory processes of Crp regulation and DNA supercoiling (22–24). However, while the specific influence of Crp on transcriptional control of genes is well documented, the extent to which unspecific or low-affinity Crp-DNA interactions directly influence global gene regulation in a NAP-like, chromatin-shaping fashion is less clear.

Several previous studies characterized the evolution of E. coli strains with deletions in the *cya* gene, which encodes the enzyme adenylate cyclase, responsible for the formation of cAMP from ATP (25–29). *cya* mutants typically experience starvation stress, as they are unable to ferment a range of Crp-dependent sugars, such as maltose and lactose. The most frequently observed compensatory mutations occur in *crp* and render Crp cAMP independent. Such Crp* mutations are easily identified by improved growth on, e.g., maltose. Here, in order to elucidate global effects of Crp inactivity, we sought to identify different types of *cya* compensatory mutations by isolating mutants that grow better at different temperatures but remain unable to efficiently ferment maltose. We sequenced the genomes of 48 mutant E. coli strains selected this way and identified new hot spot mutations that provide insights into the role and evolution of gene regulation by Crp.

## RESULTS

### Identification of two new mutational hot spots in an adenylate cyclase knockout strain by changing the selection regimen.

**FIG 1:**
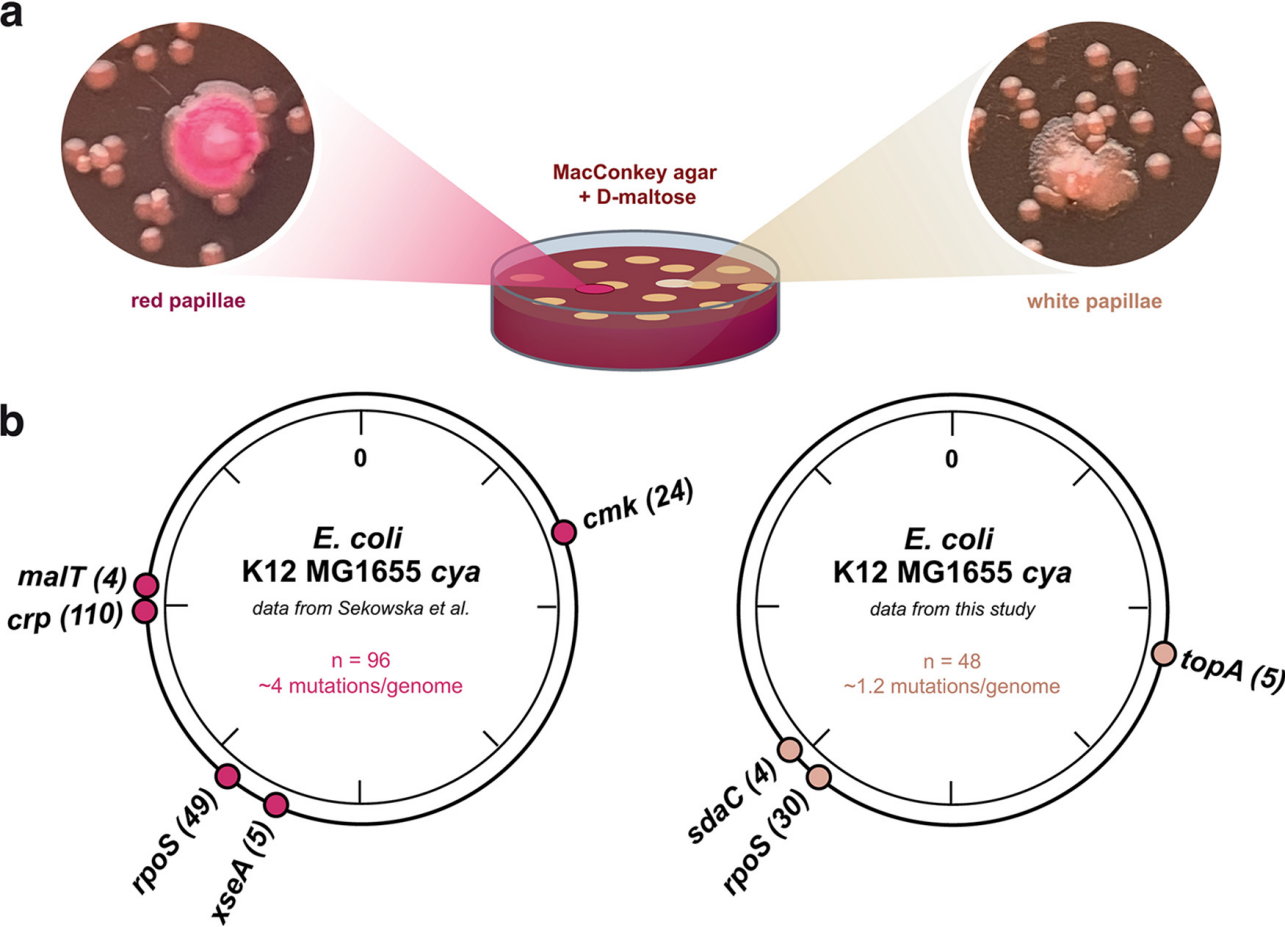
Mutational hot spots and phenotypes obtained in an E. coli adenylate cyclase mutant during evolution at different temperatures. (a) Illustration of the experimental set-up for the evolution experiment. Representative pictures of red and white papillae showing the two different phenotypes when secondary colonies develop on agar plates. (b) Schematic of mutational hot spots detected within the genome of K-12 MG1655 *cya* after prolonged incubation on maltose MacConkey plates in the study by Sekowska et al. (30) (left) and in this study (right). Numbers of identified mutations are given in parentheses. n, total number of evolved secondary colonies sequenced in the two studies.

Prolonged incubation of E. coli K-12 MG1655 *cya* colonies on maltose MacConkey agar plates at 37°C previously led to the identification of mutations in several genomic hot spots, such as *crp*, *cmk*, *rpoS*, and *malT* (30). These mutants were selected by picking secondary colonies that spontaneously outgrew their parental colonies and turned red due to maltose fermentation and a pH indicator in the agar plates (Fig. 1) (30). Mutations within the *crp* gene dominated under these conditions, leading to partial cAMP independence of the TF. However, we also observed a different colony phenotype: the formation of white secondary colonies (Fig. 1) that were more pronounced and rapidly occurring when the incubation temperature was elevated to 44°C. To explore the evolutionary response to perturbations in the cAMP-Crp global stress response in greater depth, we isolated over a period of 20 days the white secondary colonies that formed on maltose MacConkey agar at 37°C and 44°C. The isolates were restreaked several times on MacConkey agar supplemented with maltose or mannitol, and strains that were unable to efficiently ferment both sugars were selected for whole-genome sequencing (WGS).

In total, 48 strains were selected—15 isolated at 37°C and 33 isolated at 44°C—and a total of 58 mutations identified, presenting an average of only 1.2 mutations per genome (Table S1). Mutations in *rpoS* were the most frequently observed under this selection regimen, constituting 30 of the 58 mutations identified and occurring in 58% of the sequenced strains. Inactivation of RpoS happens frequently under laboratory conditions (31–33), and the majority of mutations identified in *rpoS* here were also frameshift mutations. Two new mutation hot spots, defined as a mutation that occurred more than once and had not been previously reported, were identified in the *topA* gene (5 mutations) and in an intergenic region between *ygdH* and *sdaC* (4 mutations) (Fig. 1b). The latter mutations aligned within the promoter region of the *sdaC* gene, encoding a serine-H^+^ symporter that is predominantly used to import l-serine utilized for an energy-providing role (34, 35). While these *cis*-acting mutations presumably enable improved utilization of an alternative carbon/energy source, the mutations identified here in the *rpoS* and *topA* gene are likely to have a more global effect on gene regulation and were therefore further investigated.

10.1128/mBio.02028-21.3 TABLE S1 Mutations identified in 48 isolated clones by whole-genome sequencing. Download Table S1, XLSX file, 0.03 MB. Copyright © 2021 Heyde et al. 2021 Heyde et al. https://creativecommons.org/licenses/by/4.0/ This content is distributed under the terms of the Creative Commons Attribution 4.0 International license.

In 24 of the isolates, we identified only a single mutation; 19 of these were *rpoS* mutations and two were in *topA*, suggesting that single mutations in these loci are causative for the phenotype. Thus, this different approach toward selecting *cya* compensatory mutants results in the identification of fewer genomic mutations and new mutational hot spots.

### *trans*-acting mutations that compensate for the inactivity of the global regulator Crp dominate.

In contrast to the rarely occurring *cis-*acting mutations, *trans*-acting mutations in global regulators such as Crp and the stress sigma factor RpoS are highly dominating. The so-called Crp* mutations, active in the absence of cAMP, were previously identified in 94 of 96 sequenced genomes, selected as red papillae outgrowing the initially formed colonies on maltose MacConkey agar (30). Here, by selecting only white papillae (Fig. 1), which were unable to efficiently ferment maltose and mannitol, we completely removed Crp* mutants from the observed evolutionary solution space.

**FIG 2:**
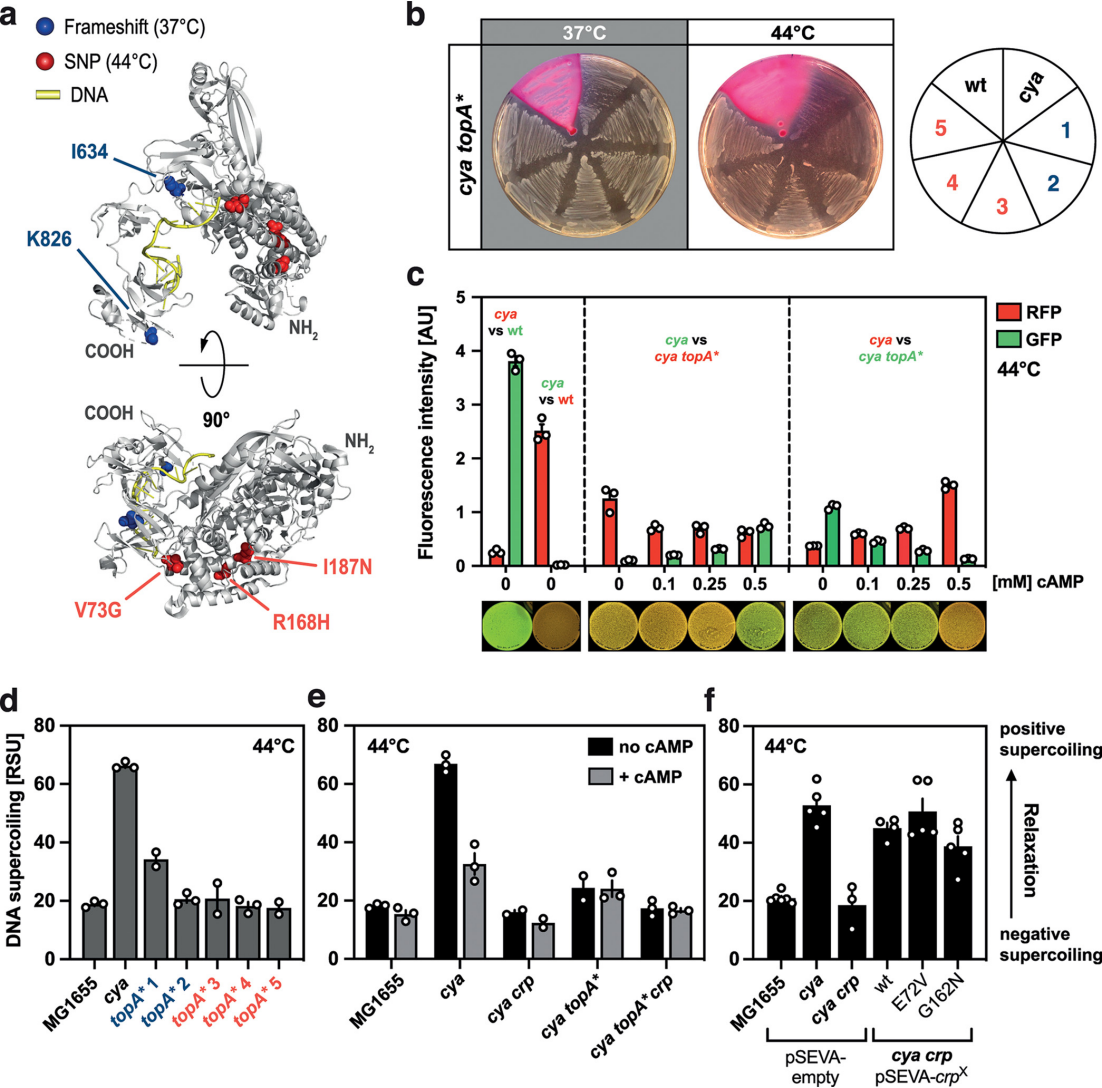
Characterization of *trans*-regulatory mutations in the *topA* gene. (a) Structural representation of topoisomerase I (gray, cartoon) bound to DNA (yellow) (Nucleic Acid Database [NDB] no. NA3316) (59). Mutated residues are indicated together with the experimental condition they were isolated under: blue, frameshift mutations isolated in papillae evolved at 37°C; red, single nucleotide variations leading to the indicated amino acid changes evolved at 44°C. (b) Growth phenotypes of the five evolved cya topA* mutant strains (1 to 5), the parental *cya* strain, and wild-type K-12 MG1655 on maltose MacConkey agar at 37°C and 44°C. (c) Growth competition assay on SM agar plates comparing *cya* and wild-type (wt) strains and *cya* and cya topA* 4 strains. All strains carry the same plasmid encoding either *gfp* (green) or *rfp* (red) under the control of a constitutive promoter to discriminate between the different strains. cAMP was supplemented in the agar at the concentrations indicated. Cultures were grown individually in liquid growth medium, adjusted for cell density, and mixed (as indicated) prior to plating and incubation for 24 h at 44°C (the bottom row shows representative pictures of fluorescence on plates). (d) Relative DNA supercoiling levels in five isolated cya topA* strains (1 to 5) compared to the parental *cya* strain and K-12 MG1655 wild type in stationary phase at 44°C using a previously described *in vivo* supercoiling reporter plasmid (38). (e) Relative DNA supercoiling levels of K-12 MG1655 wild type, *cya*, *cya crp*, cya topA*, and cya topA* *crp* and the effect of cAMP addition in stationary phase at 44°C. (f) Relative DNA supercoiling levels of the *cya crp* strain when supplemented with a plasmid expressing either the wild-type Crp protein or Crp mutants carrying the amino acid substitution E72V or G162N, as indicated, an empty vector control (pSEVA-empty), and the wild type and the ancestral *cya* strain carrying the pSEVA-empty vector control. All measurements were performed in stationary phase at 44°C. DNA relaxation increases in the direction of the arrow on the right. RSU, relative supercoiling units. All measurements were performed in biological triplicate; error bars indicate standard errors of the means (SEM).

A surprising new mutational hot spot found under our new selection regimen is *topA.* To understand the mutations obtained in the *topA* locus, we first mapped the alterations onto the crystal structure of the 865-amino-acid E. coli DNA topoisomerase I (TopoI) enzyme (Fig. 2a). Mutations isolated from papillae evolved at 37°C caused frameshifts close to the C-terminal end of the protein hosting the DNA binding domain. Mutations isolated at 44°C, on the other hand, were single amino acid substitutions in domains I (V73G) and IV (R168H and I187N) of the enzyme. The latter mutations were located close to a recently described R168 mutation that was shown to cause a >80-fold loss of relaxation activity and that was found to be crucial to hold the DNA substrate in proper conformation for cleavage and religation (36, 37). These findings point toward reduced TopoI function in the evolved cya topA* strains compared to the ancestral *cya topA*^+^ strain.

To explore the growth and relative fitness of the topA* mutations, we first compared the growth of *topA* mutants to the wild type and the parental *cya* strain on maltose MacConkey agar (Fig. 2b) at both 37°C and 44°C. In four of the five cases, mutations in *topA* resulted in increased growth relative to the *topA*^+^ *cya* ancestral strain. The exception was the cya topA* mutant strain 1 (Fig. 1b), which harbors an additional mutation in the *gyrA* gene (Table S1). To explore whether the mutations in the *topA* locus enable growth on maltose present in the plates, cya topA* mutant strains were restreaked on MacConkey medium without maltose and incubated at both 37°C and 44°C. At both temperatures, though most pronounced at 44°C, the evolved cya topA* mutants grew better than the parent *cya* strain, confirming the independence of the observed growth benefit of the sugar provided during the evolution experiment (Fig. S1). Next, competition between the wild type and the *cya* strain and between the *cya* and cya topA* strains were assayed: two strains, distinguished by expressing either *gfp* or *rfp*, were mixed at the same optical density and plated on minimal medium supplemented with maltose and different concentrations of cAMP. Following overnight incubation at 44°, cells were harvested, and relative growth estimated by quantifying green fluorescent protein (GFP) and red fluorescent protein (RFP) fluorescence in a microtiter plate reader. While the wild type, as expected, outcompeted the *cya* mutant, the cya topA* mutant strain 4 (R168H) strain likewise outcompeted the ancestral *cya* strain. This fitness benefit of cya topA* over the parental *cya* strain could, however, be reversed by supplementation of cAMP (Fig. 2c). These observations point toward a compensatory effect of *topA* mutations in a *cya* background in our experimental evolution setup.

10.1128/mBio.02028-21.1 FIG S1 Characterization of topA* mutants. Growth phenotypes of the five evolved cya topA* mutant strains (1 to 5), the parental *cya* strain, and wild-type K-12 MG1655 on MacConkey agar plates without (top) and with (bottom) 1% maltose supplementation at 37°C and 44°C. Download FIG S1, TIF file, 2.7 MB. Copyright © 2021 Heyde et al. 2021 Heyde et al. https://creativecommons.org/licenses/by/4.0/ This content is distributed under the terms of the Creative Commons Attribution 4.0 International license.

### Effect of *topA* and *crp* mutations on the supercoiling status of the cell.

Knowing that *topA* is expressed under heat shock conditions (16) and observing that *topA* mutations compensate for the lack of cAMP, we sought to investigate the consequences of *topA* mutations found during the evolution experiment on the catalytic activity of the enzyme using an *in vivo* supercoiling genetic reporter (38). The reporter plasmid carries both a constitutive promoter driving *tdtomato* expression and the supercoiling-sensitive *rdsA* (regulator of DNA supercoiling) promoter controlling *gfp*. In all cases, the evolved cya topA* strains, transformed with the reporter plasmid, showed reduced TopoI activity, resulting in an increase in negative supercoiling, compared to the parental *cya topA^+^* strain (Fig. 2d). For comparison, wild-type E. coli was included in the analysis and showed supercoiling levels similar to the cya topA* mutants. This suggests that one way in which topA* mutations compensate for the lack of cAMP is by decreasing stress-induced positive supercoiling in the cell. In line with this, addition of cAMP restored supercoiling to the wild-type level (Fig. 2e).

We additionally examined supercoiling in a *crp cya topA^+^* background, to investigate whether the presence of Crp has an impact on the structure of DNA in the absence of cAMP. The *crp* deletions were created by recombineering in the *cya* and the evolved cya topA* strains (mutant strain 4 [R168H]). Surprisingly, plasmid relaxation was lower in the *cya crp* strain than in the *cya crp^+^ topA^+^* background and was comparable to the supercoiling level observed in the wild type (Fig. 2e). Reintroduction of the *crp* wild-type gene into the *cya crp* background on a low-copy-number plasmid (pSEVA-*crp*) to complement the *crp* gene deletion reversed the observed decrease in positive supercoiling to the *cya crp*^+^ level, as did two Crp mutants carrying the amino acid substitutions E72V and G162N (Fig. 2f). While E72V is located within the cAMP binding domain of Crp and renders it unable to associate with its effector molecule needed for specific DNA binding in promoters, G162N is located within a surface loop of Crp interacting with the RNA polymerase and therefore interferes specifically with promoter activation but not binding (28, 29). This suggests that apoCrp plays a role in determining the structure of DNA in the cell.

### Carbon starvation during heat stress triggers global transcriptional changes.

**FIG 3:**
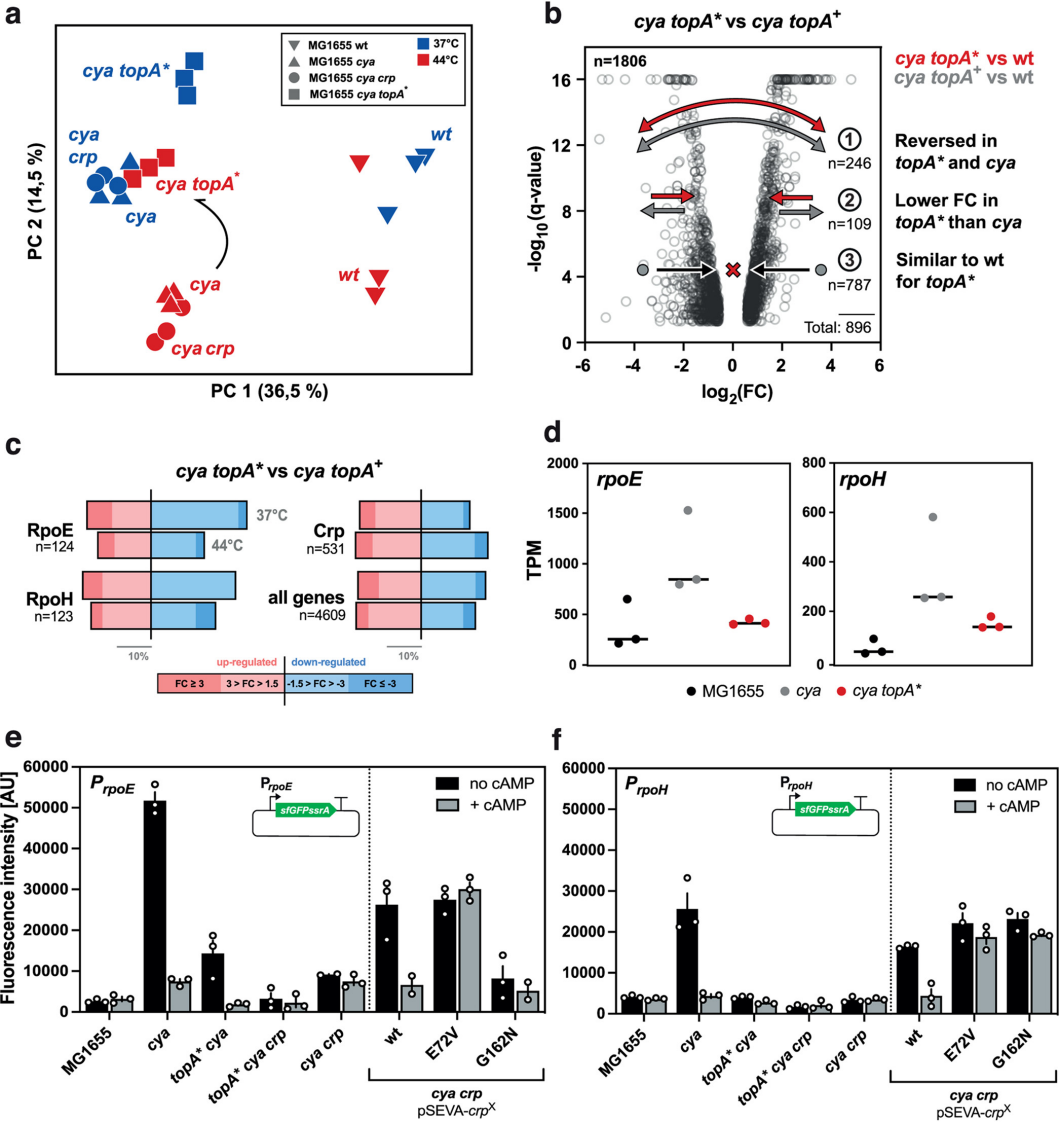
Global gene expression analysis of topA* and *crp* mutants. (a) Principal-component analysis (PCA) of transcriptome sequencing (RNA-seq) data from wild-type K-12 MG1655 and the *cya*, *cya crp*, and cya topA* mutants isolated from stationary-phase cultures at 37°C or 44°C. (b) Volcano plot of all 1,806 differentially expressed genes in the cya topA* and *cya topA^+^* data sets at 44°C. Arrows indicate changes in the differential expression pattern of the following 3 gene groups: 1, genes downregulated in the *cya* versus wild-type strain but upregulated in the cya topA* versus wild-type strain or vice versa; 2, genes that show a lower FC in expression in the cya topA* strain versus the wild type than in the *cya* strain versus the wild type; 3, genes differentially expressed only in the cya topA* versus the *cya topA^+^* mutant but not differentially expressed in the cya topA* mutant versus the wild type. FC, fold change; n, absolute number of genes found within the group. (c) DEGs in the RpoE, RpoH, and Crp regulons (as defined by regulonDB [60]; accessed 27 October 2020), as well as all genes investigated in this study as internal control comparing the cya topA* to the *cya topA^+^* mutant at both 37°C and 44°C. Only the fraction of DEGs in each regulon is displayed. Upregulation (pink) is shown on the left; downregulation (blue) is on the right. (d) Transcript-per-million (TPM) values for *rpoE* and *rpoH* in the RNA-seq data set at 37°C for the wild-type, *cya*, and cya topA* strains. (e) *In vivo rpoE* GFP reporter data for different strains (*x* axis) with or without addition of cAMP. (f) *In vivo rpoH* GFP reporter data for strains (*x* axis) with or without addition of cAMP. Data in panels e and f are from stationary-phase cultures at 44°C.

Crp and topoisomerase activities appear to be closely linked, and Crp has repeatedly been suggested to participate in the spatiotemporal organization of the bacterial genome (3, 8, 9). To explore this connection on a systems level, we analyzed the global changes in gene expression caused by the *cya-*compensatory topA* mutations by transcriptomics. Triplicate cultures of the evolved cya topA* strain (mutant strain 4), the parental *cya* strain, and the wild-type and *cya crp* strains were harvested in late stationary phase and sent for RNA extraction and sequencing by a commercial vendor. Principal-component analysis (PCA) of the total transcriptomics data confirmed reproducibility between the triplicate samples and illustrates general trajectories in the evolution of the different strains. For each strain, the data cluster differently depending on the temperature (Fig. 3a). Data from the evolved cya topA* strain locate it far from the unevolved *cya* strain at 44°C but close to *cya* samples isolated at 37°C, indicating systemic compensation of gene expression through the acquired *topA* mutation.

Different models can be proposed to explain the basis of the compensatory effect of the *topA* mutation. The global DNA supercoiling changes caused by topA* could either (i) up- or downregulate the expression of specific genes needed to reinstate/support growth in the *cya* background or (ii) enhance overall gene expression across the genome to compensate for the unavailability of active cAMP-Crp complexes. To explore these hypotheses, we carried out differential expression analysis. Transcript levels were determined using the CLC Genomics Workbench suite, and changes associated with a *P* value of ≤0.01 and changes of >1.5- and <−1.5-fold were considered to indicate significantly different expression. At 44°C, comparing the cya topA* strain with the *cya topA^+^* strain, we identified a total of 1,809 differentially expressed genes (DEGs) (Fig. 3b)—almost half of the genes in E. coli. We next explored how expression of these DEGs compared to wild-type levels and grouped them into those that were (i) downregulated in the *cya* strain versus the wild type but upregulated in the cya topA* strain versus the wild type or vice versa, (ii) less differentially expressed in the cya topA* strain versus the wild type than in the *cya* strain versus the wild type, or (iii) differentially expressed only in the cya topA* strain versus the *cya topA^+^* strain (Fig. 3b). This analysis showed that 896 of the DEGs represented reversions in the cya topA* strain toward more wild-type-like expression levels, suggesting that topA* mutations cause a major rewiring of global transcription in stationary phase to compensate for the lack of cAMP.

We further investigated DEGs in the cya topA* strain versus the *cya* strain at the two temperatures by grouping them according to their regulation by Crp or the heat stress sigma factors E and H. More Crp-regulated genes were differentially expressed in the cya topA* strain at 44°C than at 37°C (Fig. 3c), again indicating that topA* compensates for the missing cAMP at the elevated temperature. One Crp-regulated gene whose expression was consistently different between wild-type and *cya* strains but similar between wild-type and cya topA* strains was *rmf*, encoding a ribosome modulator factor (Table S2). We used this gene to validate the transcriptomics data set, by constructing an *in vivo* genetic reporter, fusing the *rmf* promoter region with *gfp* on a plasmid, and monitoring fluorescence under different growth conditions. Both in liquid culture and on agar plates, *gfp* levels from this reporter plasmid were high in the wild-type strain and in cya topA* strains but low in the *cya* mutant (Fig. S2). This represents an independent demonstration of a Crp-regulated gene that is reversed to wild-type expression level by a topA* mutation.

10.1128/mBio.02028-21.2 FIG S2 Characterization of *rmf* expression in different strain backgrounds. (a) Transcript-per-million (TPM) values for *rmf* RNA detected in the RNA-seq analysis conducted in this study. RNA was extracted from stationary-phase cultures of the wild-type, *cya*, and cya topA* strains grown at 37°C. (b) Relative fluorescence intensity of the cya topA* mutant strain and control strains carrying an *rmf* reporter plasmid (illustrated) in which *gfp* expression is under the control of the *rmf* promoter. Download FIG S2, TIF file, 2.3 MB. Copyright © 2021 Heyde et al. 2021 Heyde et al. https://creativecommons.org/licenses/by/4.0/ This content is distributed under the terms of the Creative Commons Attribution 4.0 International license.

**FIG 4:**
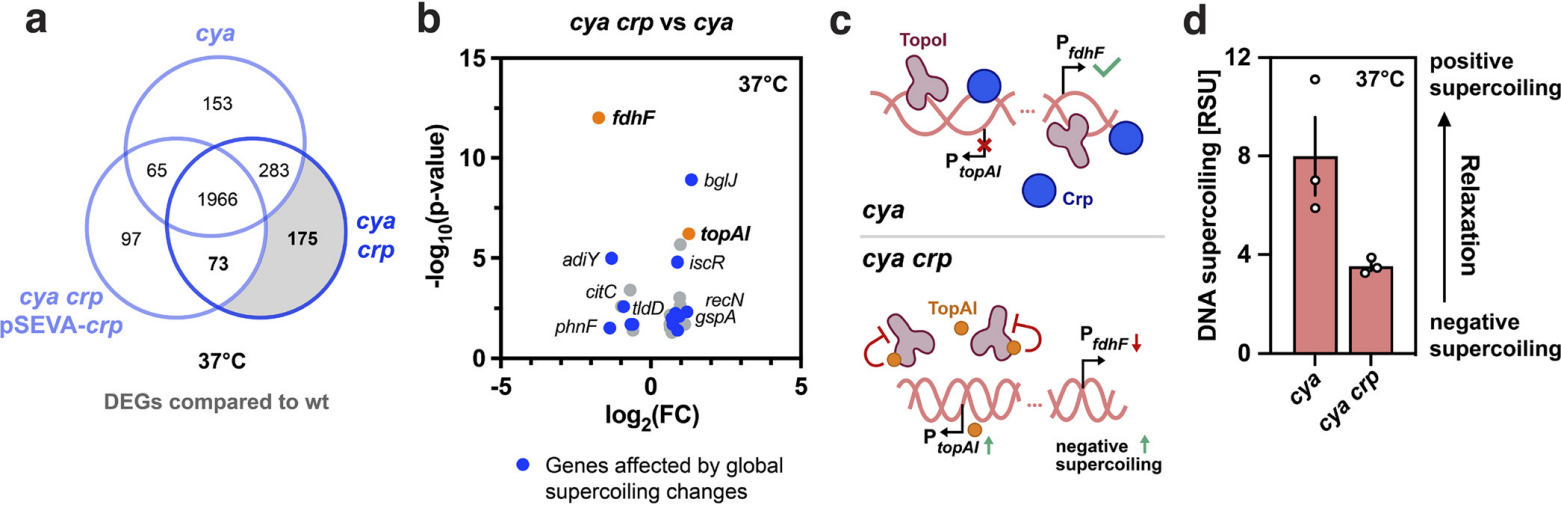
Effects of apoCrp on global gene expression explored in *cya* and *cya crp* mutant strains. (a) Venn diagram of DEGs in the *cya* and *cya crp* mutants and the *cya crp* strain complemented with wild-type Crp via the low-copy-number plasmid vector pSEVA-*crp* relative to K-12 MG1655 (wild type) under standard growth conditions (stationary phase, 37°C). Genes differentially expressed only in the *cya crp* mutant versus the wild type but not in the *cya* mutant versus the wild type or the *cya crp* pSEVA-*crp* strain versus the wild type are highlighted in light gray. (b) Volcano plot of the DEGs identified in panel a (light gray) to be uniquely differentially expressed in the *cya crp* strain compared to the wild type. Here, the same genes are plotted comparing their expression levels in the *cya crp* strain versus the *cya* strain (*n* = 30). DEGs that are affected by the global supercoiling state of the cell are in blue (*n* = 14). The supercoiling-related genes *fdhF* and *topAI* are in orange. (c) Illustration of the connection between *topAI* and *fdhF* expression in connection with the supercoiling state of the bacterial nucleoid and the availability of apoCrp. (d) Relative DNA supercoiling levels of the *cya* and *cya crp* strains in stationary phase under standard growth conditions at 37°C.

10.1128/mBio.02028-21.4 TABLE S2 Changes in gene expression in different mutants and conditions identified by transcriptomics. Raw data were sorted in sheets as indicated in the text and the legends for Fig. 3 and 4. Download Table S2, XLSX file, 0.3 MB. Copyright © 2021 Heyde et al. 2021 Heyde et al. https://creativecommons.org/licenses/by/4.0/ This content is distributed under the terms of the Creative Commons Attribution 4.0 International license.

### *rpoH* and *rpoE* are more highly expressed in the *cya* mutant, but expression is reversed to wild-type levels by topA* and *crp* mutations.

In contrast to Crp-regulated genes, such as *rmf*, the transcriptomics data showed that more RpoH- and RpoE-regulated genes were downregulated in the cya topA* strain at 37°C than at 44°C (Fig. 3c). Both RpoE and RpoH are known to be essential during heat stress and are controlled via cAMP-Crp (39, 40). To study in more detail the effect of the topA* mutation on *rpoE* and *rpoH* expression, we compared their total transcript levels (transcripts per million [TPM]) in *cya topA^+^*, cya topA*, and wild-type strains and found that they were increased in the *cya* background but closer to the wild-type level in the cya topA* strain (Fig. 3d). To validate this finding with more dynamic *in vivo* genetic reporter systems, we constructed two plasmids hosting the *rpoE* or *rpoH* promoter driving expression of *gfp.* Fluorescence levels were highly elevated for both promoters under heat stress conditions (44°C) in *cya* compared to all other strains, and this effect disappeared when cAMP was supplied in the growth medium (Fig. 3e and f). This confirms high-level expression of both *rpoH* and *rpoE* as major perturbations in the cAMP synthesis-deficient mutant, which is compensated for by mutations in *topA* and suggests that the supercoiling status of the cell significantly affects expression of the two sigma factors.

Inspired by the observation that apoCrp affects the supercoiling status of the cell, we additionally monitored the behavior of the *rpoH* and *rpoE* reporter plasmids in the *cya crp* mutant strain. In this strain, at 44°C, the fluorescence from the *rpoE* reporter was only slightly elevated compared to that in the wild-type strain (Fig. 3e), and for *rpoH*, levels were not significantly different from those in the wild type (Fig. 3f). As expected, fluorescence levels in the *cya crp* strains were not affected by addition of cAMP. This provides independent confirmation of a role for apoCrp in global gene regulation: deletion of the *crp* gene seems to reinstate wild-type-level expression of the *rpoE* and *rpoH* promoters similarly to the way higher levels of negative supercoiling facilitate this via the mutant TopoI* variants. Complementation of the *crp* deletion in the *cya crp* mutant with either pSEVA-*crp* or the two inactive Crp mutants increased expression of the *rpoH* and *rpoE* reporters, again indicating a regulatory role for apoCrp.

### Global analysis of expression of genes affected by apoCrp.

The reappearing pattern of different behavior of the *cya* and *cya crp* strains prompted us to look further into differences in gene expression in these two strains. We focused on the data obtained from stationary-phase cultures grown at 37°C, as expression changes potentially caused by apoCrp and related chromosomal rearrangements could be overshadowed by the global heat stress response at 44°C, and we wanted to depict genome-wide effects under standard conditions to understand the role of Crp as a potential nucleoid-associated protein. One hundred seventy-five genes were found to be significantly differentially expressed in the *cya crp* mutant compared to the wild type, but not when either the *cya* mutant and the wild type or the *cya crp* strain complemented with apoCrp (via pSEVA-*crp*) and the wild type were compared (Fig. 4a). We selected these genes in a comparison of the *cya crp* and *cya* data sets and plotted their distribution in a volcano plot to visualize the DEGs (Fig. 4b). Interestingly, among the 30 genes found to be significantly differentially expressed between the *cya crp* and *crp* strains, 14 genes (Fig. 4b, blue) are either directly or indirectly (Table S3) affected by the supercoiling state of the bacterial genome, pointing again toward global supercoiling-related changes in the *cya crp* strain. Within the DEGs identified from the data for *cya crp* strain versus the wild type, two supercoiling-related genes stood out: (i) *topAI*, encoding an inhibitor of TopoI (41), was among the highest upregulated genes, and (ii) *fdhF*, encoding E. coli formate dehydrogenase H previously reported to be upregulated upon gyrase inhibition (42) (i.e., downregulated by negative supercoiling), was among the most significantly downregulated genes. This suggests increased negative supercoiling upon removal of apoCrp, since upregulation of the TopoI inhibitor TopAI leads to decreased TopoI activity, which would result in more negative supercoiling and reduced expression from supercoiling sensitive genes such as *fdhF* (Fig. 4c). This is in line with the supercoiling reporter plasmid data, as plasmid DNA was 2-fold less negatively supercoiled at 37°C in the *cya* strain than the *cya crp* strain (Fig. 4d).

10.1128/mBio.02028-21.5 TABLE S3 Changes in gene expression in different mutants identified by transcriptomics. Raw data were sorted as indicated in the text and the legend for Fig. 4b. Download Table S3, XLSX file, 0.01 MB. Copyright © 2021 Heyde et al. 2021 Heyde et al. https://creativecommons.org/licenses/by/4.0/ This content is distributed under the terms of the Creative Commons Attribution 4.0 International license.

## DISCUSSION

It is striking how mutations in global gene regulation dominate the picture in experimental evolution. RpoS and Crp* mutations have been described numerous times, but to our knowledge, the current study is the first of its kind reporting that *topA* mutations compensate for loss of the global stress signal cAMP. On the other hand, supercoiling has previously been suggested to have broader significance in stress-induced transcription (43, 44), supported by experimental evolution experiments that demonstrated the importance of DNA topology as a global factor in bacterial gene regulation and its sensitivity to adaptive mutations. In the long-term-evolution experiment reported by Crozat et al., parallel changes in topology were detected in 10 out of 12 initially identical populations propagated in a defined environment for 20,000 generations (45). The level of DNA supercoiling was increased due to mutations in the genes of the global regulators *topA* and *fis*. Competition assays showed that both mutations caused direct and substantial fitness benefits. Further investigations of the ancestor and evolved clones from all 12 populations showed high levels of molecular and genetic parallelism, harboring mutations in multiple genes involved in DNA supercoiling (46). Several other experiments conducted in E. coli under different selection regimens found mutations in the *topA* gene, further emphasizing the importance of selection of DNA topology traits (47–50).

Recently, point mutations in the *topA* gene were described as giving rise to mutator phenotypes influencing the mutational spectrum of E. coli to allow fast emergence of drug resistance genotypes (36). In that study, a single substitution in the conserved residue R168C in TopoI resulted in an accelerated rate of tandem duplications, known to be a source of genetic instability and to play a significant role in bacterial evolution. Here, we identified a different mutation (R168H) in the same position in TopoI, with a major effect on global gene regulation. These findings point toward a new class of fitness-enhancing mutations and confirm that the control of DNA supercoiling is a hot spot for selection in evolving bacterial populations.

Considering the frequency of observed mutations related to DNA topology in experimental evolution and the proposed role of Crp as a nucleoid-associated protein (3, 8, 9), our finding that *topA* mutations compensate for the lack of cAMP is not surprising. We found that almost half of the genes in E. coli change expression when the *cya* mutant was compared with the cya topA* mutants, which have restored growth and expression of many genes back to wild-type levels. A simple interpretation of this observation is that activator-independent activity of many of these activator-dependent promoters is increased by extra negative supercoiling. Among these genes are global factors such as the sigma factor-encoding *rpoH* and *rpoE*, and *rmf* encoding a ribosome modulator factor. Our suggestion that the supercoiling status of the cell influences expression of these factors is supported by earlier findings that showed supercoiling to affect the promoter activity of *rpoH* (12) and that expression of *rmf* is growth phase dependent (51), a process directly influenced by supercoiling itself (52). To our knowledge, no direct connection between *rpoE* expression and the supercoiling state of the cell has been reported so far, while our data strongly suggest such a connection.

It is interesting that we also isolated a strain with a single mutation in the genome in the gene encoding the FtsH protease, which is involved in controlling the stability of RpoH (13). Thus, it is possible that this *ftsH* mutant counteracts the perturbed expression of *rpoH* in the *cya* strain. The simultaneous occurrence of a topoisomerase and a gyrase mutation in another of the evolved strains strongly suggests a complete loss of TopoI function in this mutant. Complete loss-of-function mutations in *topA* are lethal for E. coli but can be compensated for by a *gyrA* deletion, creating a viable but growth-impaired strain (53, 54).

Crp was previously suggested to represent an example of a TF that has evolved from a NAP (3), supported by circular dichroism, sedimentation analysis, and electron microscope studies showing cooperative DNA binding and condensation by apoCrp (6, 7). In this model, Crp evolved from being a NAP with the role of shaping the genome by unspecific binding to binding more frequently in proximity to promoters and assisting in recruiting the RNA polymerase. However, this model did not discuss the role of the cAMP ligand and how this allosteric control mechanism entered the picture in evolution. The different effects of apoCrp observed here expand this model, suggesting that during evolution, apoCrp played a NAP-like role that preceded both the role of cAMP as an allosteric regulator and the binding to high-affinity sites in the DNA. This model is in good agreement with the paradoxical observation that Crp homologs exist in bacteria like Pseudomonas aeruginosa that contain little or no cAMP (55). Possibly, the sensing of cAMP and the role of Crp in carbon catabolite repression is a special case in the model bacterium E. coli and homologs more commonly play NAP-like roles in the bacterial kingdom.

## MATERIALS AND METHODS

### Bacterial strains.

All experiments were performed using E. coli strain K-12 MG1655 *fnr cya* (*cya*::*cat* Δ*fnr*) described in Sekowska et al. (30). The strain harbors a deletion of the *cya* gene introduced via λRed-induced recombination, as well as a deletion of the *fnr* gene, a homologue of *crp.*

### Secondary-colony-formation assay.

For growth on plates, MacConkey medium was used (Difco MacConkey agar base) supplemented with 1% maltose as carbon source and 5 mg/liter chloramphenicol. To obtain isolated colonies on the plates, early stationary-phase bacterial cultures grown in liquid lysogeny broth (LB; Thermo Fisher Inc.) medium (growth for 7 h at 37°C) were diluted in sterile water containing 9 g/liter sodium chloride to a concentration of 2.5 × 10^4^ bacteria/ml, and 100 μl of the bacterial suspension was spread onto MacConkey plates containing the appropriate antibiotics. The plates were subsequently placed in closed plastic containers containing beakers with water to ensure constant humidity and incubated for the duration of the experiment at either 37°C or 44°C. Papillae were purified by restreaking 3 times on fresh plates supplemented with the same carbon source and antibiotic as in the secondary-colony-formation assay.

### Plate reader experiments.

For growth assays of evolved and nonevolved K-12 MG1655 *cya* strains, *in vivo* supercoiling assays with the supercoiling reporter plasmid pSupR (38) and *gfp* promoter reporter assays cells were inoculated from single colonies and cultured for 7 h in 5 ml liquid growth medium (LB) supplemented with 2% glucose to minimize the risk of mutations arising during the cultivation time. Dilutions of 1:50 were grown subsequently for 20 h in 96-well plates and 200 rpm at 37°C or 44°C, covered with a gas-permeable adhesive seal (Thermo Fisher Scientific, Inc.) to avoid evaporation. Optical density at 600 nm (OD_600_) and fluorescence (GFP, excitation at 485 nm and emission at 528 nm; RFP, excitation at 488 nm and emission at 588 nm) was measured at 10-min intervals with continuous shaking using a Synergy H1 plate reader (BioTek Instruments, Inc.). All measurements were performed in biological triplicate.

### Sequencing.

DNA purification for whole-genome sequencing was performed using DNeasy blood and tissue kit 50 (Qiagen, Inc.) starting with 1 ml overnight culture according to the manufacturer’s instructions. Isolated DNA was eluted twice using 200 μl 10 mM Tris (pH 8.5). The genomic libraries were generated using the TruSeq DNA HT library prep kit (Illumina Inc.). Forty-eight independent papillae were selected for whole-genome sequencing using Illumina sequencing adapters D701 (ATTACTCG) and D501 (TATAGCCT). Data analysis was performed using the breseq software developed by Deatherage and Barrick (56). Of 48 strains sequenced, 24 carried an additional mutation in an unstable IS*2* element previously shown to cause a high degree of genetic variation in different E. coli strains (57), and these mutations were excluded from the total number of mutations. For transcriptomics analysis, cells were grown in LB liquid medium until stationary phase at 37°C or 44°C. After 12 h of growth, cultures were normalized to OD_600_, and 1 × 10^7^ cells were harvested via centrifugation. Pellets were immediately frozen in liquid nitrogen.

RNA extraction, library preparation, and RNA sequencing were performed by Novogene, Inc., Cambridge, UK. All data analysis was performed using CLC Genomics Workbench 20. FastQ files were imported into CLC Genomics Workbench for data processing as paired reads. Reads were trimmed using a quality score limit of 0.05, ambiguous nucleotides (none allowed), and adapters (5′ adapter, AATGATACGGCGACCACCGAGATCTACACTCTTTCCCTACACGACGCTCTTCCGATCT; 3′ adapter, GATCGGAAGAGCACACGTCTGAACTCCAGTCACATCACGATCTCGTATGCCGTCTTCTGCTTG). All sequence-based trimming followed standard alignment settings with a mismatch cost of 2, a gap cost of 3, a minimum internal score of 10, and a minimum end score of 4. Trimmed reads were then mapped to a reference sequence (GenBank no. U00096.3) using global alignment with standard alignment settings of a match score of 1, a mismatch cost of 2, a linear gap cost of 3, and length fraction and similarity fraction at 0.5 and 0.8, respectively. Following this, the reads were examined for structural variants (quality *P* < 0.0001 and maximum number of mismatches = 3 for end breakpoints), including deletions and insertions, and, based on this information, were realigned using local realignment (two iterations; maximum guidance variant length, 200 bp). Quality distribution followed an average PHRED score of 37, and coverage was on average 99.9% with an average nucleotide distribution of 25% and GC content of ∼50%. Read counts for each gene were then converted to TPM values by the software and exported to Excel. For subsequent comparison between conditions, samples were normalized based on a statistical model, described in the software manual to consist of TMM (trimmed mean of the M values) normalization, calculation of TMM-adjusted log counts of counts per minute, and finally, applied separately for each gene, Z-score normalization (mean = 0, standard deviation = 1). Fold change (FC) values were then calculated (applying a *t* test to compare groups) and reported as log_2_(FC) for ease of comparison along with *P* values and false-discovery-rate (FDR)-adjusted *P* values (*q* values) for the statistical significance.

### Genome engineering.

To generate the *crp* knockout strains MG1655 *cya crp* and MG1655 topA* *crp* (topA* 4: R168H), cells were transformed with plasmid pSIM19 (58), encoding heat-inducible λRed recombination proteins, and recombineering was performed using a *tetA* integration cassette flanked by 50-base homology areas for the *crp* locus. Colonies were screened for the desired *crp* knockout (*crp*::*tetA*) by Sanger sequencing.

### Fitness competition assays.

To monitor fitness competition within two strains in a mixed culture, each strain was transformed with an expression vector harboring either a *gfp* or *rfp* gene under the control of a constitutive promoter. Precultures for each strain were grown individually in liquid medium for 7 h, before the number of CFU was calculated for each culture based on OD_600_. Equal CFU for each strain to be compared were subsequently mixed and plated onto simple MacConkey (SM) agar supplemented with maltose and up to 0.5 mM cAMP. SM medium composition for 300 ml was soybean peptone (5.1 g; 70178-100G; Sigma-Aldrich), protease peptone (0.9 g; P0431-250G; Sigma-Aldrich), NaCl (1.5 g; Sigma-Aldrich), and agar (4.05 g; Sigma-Aldrich). Colony formation was allowed overnight at 44°C. Colonies were subsequently harvested from the plates and resuspended in sterile 0.9% sodium solution, and the fluorescence signals for both GFP and RFP channels were measured. To control for plasmid-inflicted differences in growth, all experiments were repeated with reverse plasmid combinations.

### Data availability.

All sequencing data connected to this study have been deposited in the European Nucleotide Archive (ENA). Whole-genome sequencing data can be accessed via accession number PRJEB43656. RNA sequencing data can be accessed via accession number PRJEB43645.
