# Supplementary figures and images for: Restoring Global Gene Regulation through Experimental Evolution Uncovers a NAP (Nucleoid-Associated Protein)-Like Behavior of Crp/Cap
Source: mBio. 2021 Oct 26;12(5):e02028-21. doi: 10.1128/mBio.02028-21 (PMC8546631; doi:10.1128/mBio.02028-21)

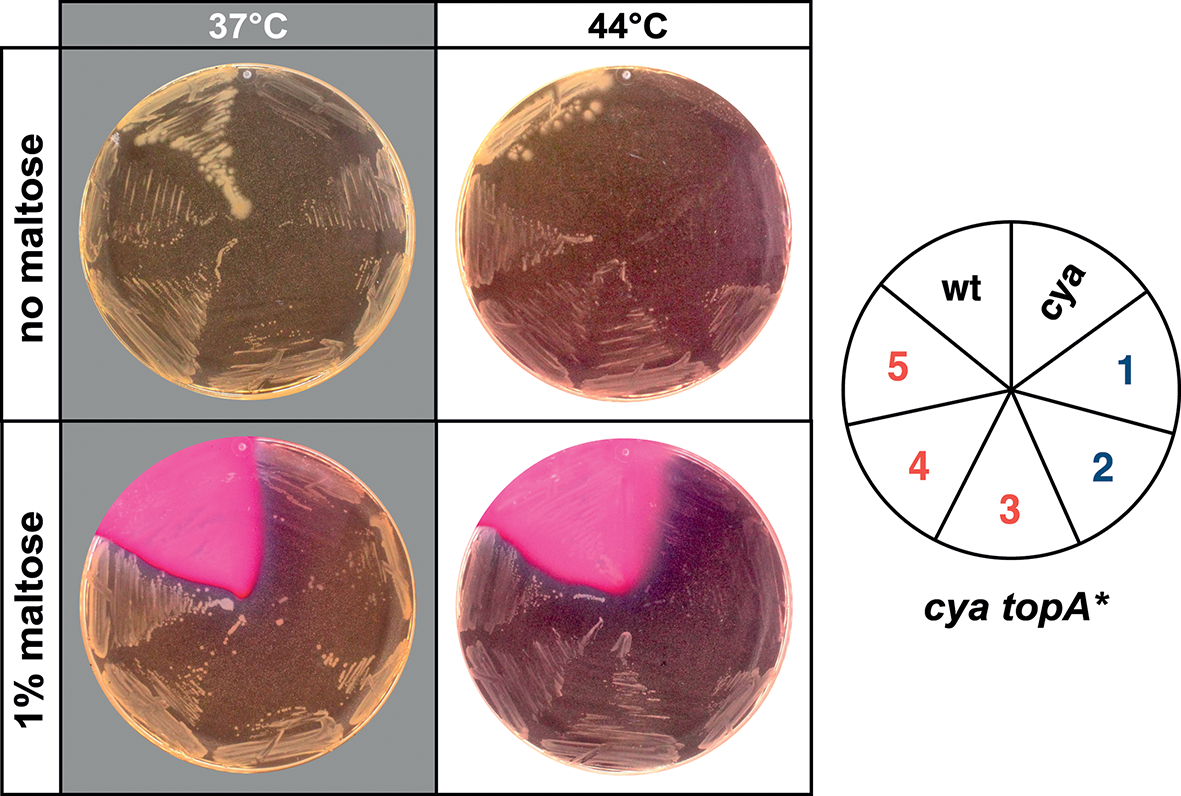

Supplement: FIG S1 [file mbio.02028-21-sf001.tif]

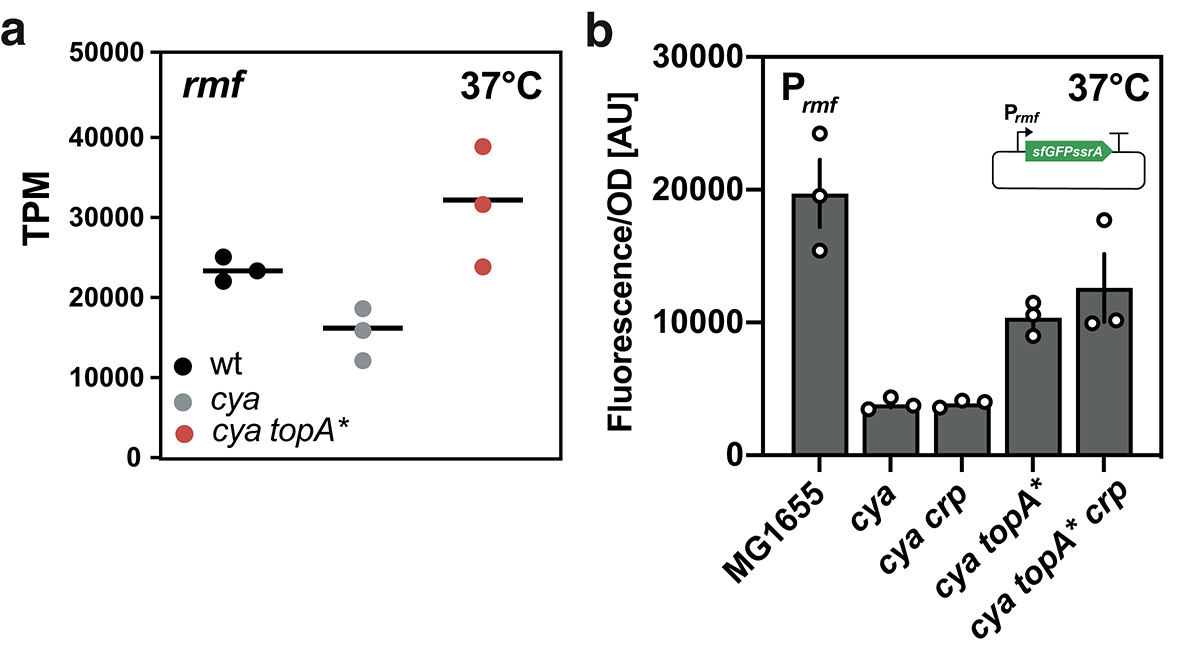

Supplement: FIG S2 [file mbio.02028-21-sf002.tif]
